# Polynomial and Wavelet-Type Transfer Function Models to Improve Fisheries’ Landing Forecasting with Exogenous Variables

**DOI:** 10.3390/e21111082

**Published:** 2019-11-05

**Authors:** Eliana Vivas, Héctor Allende-Cid, Rodrigo Salas, Lelys Bravo

**Affiliations:** 1 Escuela de Ingeniería Informática, Pontificia Universidad Católica de Valparaíso, Valparaíso 2340025, Chile; eliana.vivas.r@mail.pucv.cl; 2 Escuela de Ingeniería C. Biomédica, Universidad de Valparaíso, Valparaíso 2391415, Chile; rodrigo.salas@uv.cl; 3 Department of Statistics, University of Illinois at Urbana-Champaign, Champaign, IL 61820, USA; lbravo@illinois.edu

**Keywords:** fisheries’ landings, time series forecasting, wavelets

## Abstract

It is well known that environmental fluctuations and fishing efforts modify fishing patterns in various parts of the world. One of the most affected areas is northern Chile. The reduction of the gaps in the implementation of national fisheries’ management policies and the basic knowledge that supports the making of such decisions are crucial. That is why in this research, a transfer function method with variable coefficients is proposed to forecast monthly disembarkation of anchovies and sardines in northern Chile, taking into account the incidence of large-scale climatic variables on landings. The method uses a least squares procedure and wavelets to expand the coefficients of the transfer function. Linear estimators of the time varying coefficients are proposed, followed by a truncation of the wavelet expansion up to an appropriate scale. Finally, the estimators for the transfer function coefficients are obtained by using the inverse wavelet transformation. Research results suggest that the transfer function models with variable coefficients fit the behavior of the anchovies’ landing with great accuracy, while the use of transfer function models with constant coefficients fits sardines’ landings better. Both fisheries’ landings could be explained to a large extent from the large scale climatic variables.

## 1. Introduction

Fish are organisms that cannot regulate the temperature of the environment independently, and the environment temperature changes influence their geographical distribution, migratory routes, and occupation of habitat [1]. On the other hand, although the species present variability associated with environmental changes, the composition and abundance are also affected by predators, competitors, and prey [2]. The link between the variation of anchovy abundance and environmental changes in different time-space scales opens the possibility of predicting fluctuations in landings in the short, medium, and long term [3]; which is one of the main objectives of fisheries’ management [4].

As indicated by [5], it is fundamental to consider the impact of the environment and the interactions between fisheries for their management. In effect, the fisheries show different trends in response to environmental changes, since these changes affect various stages of larvae, reproduction, grazing habitat, and migration of different populations. In addition, an inevitable increase in fishing effort must be added. Potential climate change and climate variability at different time scales have immediate or phase effects, both locally and regionally. Possible changes in environmental variables such as sea surface temperature (SST), depth of the mixing layer, depth of the thermocline, intensities of up-welling currents, the mechanism of nutrient concentration, and changes in the ice marine layers [2], although mild, may affect the food chain, thus drastically altering the abundance, distribution, and availability of fish populations. In addition, climatic change could have consequences on the composition of the community and the performance of ecosystems [6].

Regarding the environment and resource analysis in the anchovy and sardine fisheries of northern Chile, the work in [7] developed an artificial neural network (ANN) model for the anchovies’ fishery. In [8], the authors developed a multivariate ANN model considering monthly environmental variables such as the sea surface temperature, up-welling index, and sea level; while [9] developed ANN models for anchovy and sardine, respectively, taking into account, in addition to the environmental variables, the interaction between species. These studies made a brief analysis of the correlation between variables, self-correlation, and cross-correlation using non-linear functions to find functional relationships to introduce different models [2]. On the other hand, the wok in [10] predicted the environmental variability in the anchovy fishery in the northern zone of Chile, through the development of spatio-temporal indicators of the ecosystem, statistical relationships between indicators, GIS functions (Geographical Information Systems), and ANN models, offering an integration in the prediction of anchovy abundance.

With respect to other statistical techniques implemented to forecast fishing landings, there was the application of a hybrid model studied by [11], in which the potentialities of autoregressive models integrated moving averages (ARIMA) were combined with wavelet theory to enhance the precision of fishing landings’ forecasts in Malaysia. Their study found that the combined model provided more accurate forecasts of fishing landing series than the individual ARIMA model. Other studies have presented a forecast strategy based on the decomposition of stationary wavelets combined with linear regression to improve the accuracy of pelagic one month ahead fish catches predictions of the fishing industry in the southern zone of Chile [12]. The authors demonstrated the usefulness of the strategy in the anchovy catch dataset for monthly periods, explaining 98% of the variance with a parsimonious reduction.

Considering the above and in virtue of the fact that in Chile, the average annual landings in the last 30 years was 4.8 million tons and the agricultural resources in the northern zone represent 40% [13], as well as given that in this area, the fishery is based successively on anchoveta (*Engraulis ringens*) and sardine (*Sardinops sagax*), with notable changes associated with fishing effort and environmental fluctuations (see [14,15]), it is considered pertinent to implement scientific techniques aimed at studying functional relationships that can be analyzed in depth, in order to reduce gaps in the implementation of national fisheries’ management policies and provide the basic knowledge that supports the making of such decisions [2].

Currently, the correct prediction of fishing landings in particular is a point of special interest for fisheries’ management, and researchers who focus on modeling time series of landings are looking for prediction models that take into account various patterns. In the literature, most researchers implement potential methods such as ANN and hybrid models such as autoregressive integrated mobile average with ANN, among others, effectively synced to model time series and predict fishing landings; however, there is still a wide range of hybrid techniques that can be implemented to achieve improvements in predictions.

In this sense, this research proposes the implementation of highly predictive techniques to model and study climatic phenomena, specifically the quantitative characterization of the elements that determine the monthly disembarkation of anchovies and sardines in northern Chile. The work revolves around the following question: Which time series model would allow forecasting more accurately the monthly disembarkation of anchovies and sardines registered in northern Chile, under the influence of macro-climatic variables such as the sea surface temperature and the associated ENSO phenomenon?

The benefits of our research reside in the improvements obtained in the adjustment and forecasting of anchovy landings when the series are broken down into their high and low frequency components, by expanding the transfer function coefficients to a time varying approach by using a least squares procedure. It also highlights the improved performance of using the combination of traditional statistical techniques with the aforementioned extension when implemented to forecast sardine landings. Likewise, seeking to optimize the goodness of fit and quality of the forecast, it was also observed that after the application of various transformations to stabilize the variability of the observed series, significant improvements in the results could be achieved.

The paper is divided as follows: In Section 2, we briefly describe the time series modeling strategy and the required steps to fit these models. In Section 3, we explain the datasets used in the analysis, the methodology to process them, and all the results at each step, when fitting the transfer function models. We finally provide in Section 4 some conclusions and potential extensions of this work.

## 2. Materials and Methods

### 2.1. Environmental Setting and Data

Industrial fishing in the northern part of the country began in the 1950s with landings of Peruvian anchovy (*Engraulis ringens*), which increased, fluctuated, and then fell strongly in 1972–1973, remaining low until 1985, when they again began to fluctuate and increase, reaching new historic levels [13]. After the collapse of anchovy in 1972–1973, the sardine became a targeted species (*Sardinops sagax*), with catches increasing until 1985, before falling notably and remaining low until the present. The study zone comprised the area covered by the industrial seine fishing fleet that operates in northern Chile $(18^{\circ }21^{\prime }$–$24^{\circ }00^{\prime }$ S) from the coast to $73^{\circ }$ W. The analyzed data included environmental and fishing registers for the 1963–2011 period, Table 1 shows a description of each variable considered for analysis.

### 2.2. Wavelet Transfer Function Model

There are many situations requiring the modeling of the impact of a regressor variable on a response variable through time, when the regressors and the response variables are both assumed stochastic processes. Herein, we will use the term predictors for the regressor variables and predictants for the response variable. One or more predictors can be considered as input variables to the model. On the other hand, predictors can have a lagged effect on the predictant variables, and one must decide how many past values of the predictor variable would make an impact on the predictant variable [16]. Following the transfer function models with the time varying approach used by [17], one might consider the following model:

(1) $$Y_{t,T}=\sum_{i=1}^{m}\delta_{i}\left(\frac{t}{T}\right)Y_{t-i,T}+\sum_{j=0}^{n}\omega_{j}\left(\frac{t}{T}\right)X_{\left(t-j,T\right)}+\varepsilon_{t},$$

where the time series $X_{t,T}$ and $Y_{t,T}$ correspond to the explanatory and response variable, respectively, T is the number of observations, and $\varepsilon_{t}$ is considered independent and identically distributed $\left(0,\sigma^{2}\right)$ random error. It is assumed that the error and the entries in the series are independent. The functions $\delta_{i}\left(u\right)$, *i* = 1, …, *m* and $\omega_{j}\left(u\right)$, *j* = 0, 1, …, *n*, have compact support in the interval [0,1] and are connected to the underlying series by an appropriate adjustment on the time scale, u=t/T. For the estimation of $\delta_{i}\left(u\right)$ and $\omega_{j}\left(u\right)$, *i* = 1, …, *m*, *j* = 0, 1, …, *n*, wavelet expansions are used in the time domain. The estimators of the wavelet coefficients are obtained through the least squares method [17].

From two basic functions, the scaling function ϕ(x) and the wavelet function ψ(x), infinite collections of scale and translated versions are defined, $\phi_{j,k}\left(x\right)=2^{j/2}\phi \left(2^{j}x-k\right)$, $\psi_{j,k}\left(x\right)=2^{j/2}\psi \left(2^{j}x-k\right)$, $j,k\in Z=\left\{0,\pm 1,\ldots \right\}$. It is assumed that $\phi_{\left(l,k\right)}{\left(.\right)}_{\left(k\in z\right)}$$\cup {\left\{\psi_{j,k}\left(.\right)\right\}}_{j\geq l;k\in z}$ form an orthonormal basis of $L_{2}\left(R\right)$, for some coarse scale (l). To achieve a parsimonious representation of the amplitude of wavelet function classes in the series, it is necessary to construct ϕ and ψ functions with compact support, which generate an orthonormal system, with frequency and spatial localization [17]. The functions $\omega_{j}\left(u\right)$ and $\delta_{i}\left(u\right)$ are defined in a compact range [0,1]. Therefore, an orthonormal system that spans $L_{2}\left(\left[0,1\right]\right)$ must be taken into account. Some authors use an adaptation step [18] with the periodized wavelet defined by:

(2) $$\begin{array}{cc} \; & {\tilde{\phi }}_{j,k}\left(x\right)=\sum_{n\in Z}\phi_{j,k}\left(x-n\right)\\ \; & {\tilde{\psi }}_{j,k}\left(x\right)=\sum_{n\in Z}\psi_{j,k}\left(x-n\right) \end{array}$$

and these generate a ladder at the multiple resolution level ${\tilde{V}}_{0}\subset {\tilde{V}}_{1}\subset \cdots$, in which the spaces ${\tilde{V}}_{j}$ are generated by ${\tilde{\psi }}_{j,k}$. For those that are not necessary negative values of *j*, $\tilde{\phi }={\tilde{\phi }}_{0,0}=1$. If j≤0, ${\tilde{\psi }}_{j,k}\left(x\right)=2^{\frac{-j}{2}}$ (see [19]). In the work, periodized wavelets are denoted simply by $\psi_{j,k}$. Consequently, for any function $f\in L_{2}\left(\left[0,1\right]\right)$, an orthogonal series expansion can be considered of the form:

(3) $$f\left(x\right)=\alpha_{0,0}\phi \left(x\right)+\sum_{j\geq 0}\sum_{k\in I_{j}}\beta_{j,k}\psi_{j,k}\left(x\right),$$

where we take l=0 and $I_{j}=\left\{k:k=0,\cdots ,2^{j}-1\right\}$. For each *j*, the set $I_{j}$ brings the values of *k*, so that $\beta_{j,k}$ belongs to the scale $2^{j}$. For example, for *j* = 3, there are eight wavelet coefficients on a scale of $2^{3}$. The wavelet coefficients are given by:

(4) $$\begin{array}{cc} \; & \alpha_{0,0}=\int f\left(x\right)\phi \left(x\right)dx,\\ \; & \beta_{j,k}=\int f\left(x\right)\psi_{j,k}\left(x\right)dx \end{array}$$

Often, the sum of Equation (3) is considered for a maximum level J, such that we approximate *f* in the space ${\tilde{V}}_{j}$ (for more details, see [17]).

(5) $$f\left(x\right)\approx \alpha_{0,0}\phi \left(x\right)+\sum_{j=0}^{J-1}\sum_{k\in I_{j}}\beta_{j,k}\psi_{j,k}\left(x\right),$$

#### Estimators of Time Varying Coefficients

The objective is to estimate the functions $\delta_{i}\left(u\right),i=1,2,\cdots m$ and $\omega_{j}\left(u\right),j=1,2,\cdots n$$(\delta_{i}\left(u\right)$ and $\omega_{j}\left(u\right)\in \left[0,1\right])$ that appear in model Equation (1), given the T observations of the series. We assume that the orders of m and n are fixed and known. The idea is to expand these functions in wavelet series of the form [17]:

(6) $$\begin{array}{cc} \; & \delta_{i}\left(u\right)=a_{0,0}^{\left(\delta_{i}\right)}\phi \left(u\right)+\sum_{j=0}^{J-1}\sum_{k\in I_{j}}\beta_{jk}^{\left(\delta_{i}\right)}\psi_{jk}\left(u\right)\\ \; & \omega_{i}\left(u\right)=a_{0,0}^{\left(\omega_{i}\right)}\phi \left(u\right)+\sum_{j=0}^{J-1}\sum_{k\in I_{j}}\beta_{jk}^{\left(\omega_{i}\right)}\psi_{jk}\left(u\right) \end{array}$$

The empirical wavelet coefficients are obtained by minimizing the expression:

(7) $$\sum_{t=v+1}^{T}{\left(Y_{t,T}-\sum_{i=1}^{m}\delta_{i}\left(u\right)Y_{t-i,T}-\sum_{j=0}^{n}\omega_{j}\left(u\right)X_{t-j,T}\right)}^{2}$$

$\delta_{i}\left(u\right)$ and $\omega_{j}\left(u\right)$ are replaced by Equation (6) for $v=max\left(m,n\right)$. In matrix notation, the solution of the least squares problem given by Equation (7), for 0≤m≤J−1, is obtained from the equations:

(8) $$\left[\begin{array}{c} {\hat{\beta }}^{\left(\delta_{1}\right)}\\ {\hat{\beta }}^{\left(\omega_{0}\right)} \end{array}\right]={\left[\begin{array}{cc} \Psi_{Y}^{{}^{\prime }}\Psi_{Y}\; & \Psi_{Y}^{{}^{\prime }}\Psi_{Y}\;\\ \Psi_{X}^{{}^{\prime }}\Psi_{Y}\; & \Psi_{Y}^{{}^{\prime }}\Psi_{X} \end{array}\right]}^{-1}\left[\begin{array}{c} \Psi_{Y}^{{}^{\prime }}Y\\ \Psi_{X}^{{}^{\prime }}X \end{array}\right]$$

where you have to do:

(9) $$\begin{array}{cc} \; & \Psi_{Y}=\left[\begin{array}{ccccc} \Phi_{Y}\; & \Psi_{Y}^{0} & \Psi_{Y}^{1} & \cdots & \Psi_{Y}^{\left(J-1\right)} \end{array}\right];\\ \; & \Psi_{X}=\left[\begin{array}{ccccc} \Phi_{X}\; & \Psi_{X}^{0} & \Psi_{X}^{1} & \cdots & \Psi_{X}^{\left(J-1\right)} \end{array}\right] \end{array}$$

(10) $$\Phi_{Y}=\left[\begin{array}{c} \phi_{0,0}\left(\frac{2}{T}\right)Y_{1,T}\\ \phi_{0,0}\left(\frac{3}{T}\right)Y_{2,T}\\ \vdots \\ \phi_{0,0}\left(\frac{T}{T}\right)Y_{T-1,T} \end{array}\right];\Phi_{X}=\left[\begin{array}{c} \phi_{0,0}\left(\frac{2}{T}\right)X_{1,T}\\ \phi_{0,0}\left(\frac{3}{T}\right)X_{2,T}\\ \vdots \\ \phi_{0,0}\left(\frac{T}{T}\right)X_{T-1,T} \end{array}\right]$$

(11) $$\Psi_{Y}^{\left(m\right)}=\left[\begin{array}{cccc} \psi_{m0}\left(\frac{2}{T}\right)Y_{1,T}\; & \psi_{m1}\left(\frac{2}{T}\right)Y_{1,T} & \cdots & \psi_{m,2^{m}-1}\left(\frac{2}{T}\right)Y_{1,T}\\ \psi_{m0}\left(\frac{3}{T}\right)Y_{2,T} & \psi_{m1}\left(\frac{3}{T}\right)Y_{2,T} & \cdots & \psi_{m,2^{m}-1}\left(\frac{3}{T}\right)Y_{2,T}\\ \vdots & \vdots & \ddots & \vdots \\ \psi_{m0}\left(\frac{T}{T}\right)Y_{T-1,T} & \psi_{m1}\left(\frac{T}{T}\right)Y_{T-1,T} & \cdots & \psi_{m,2^{m}-1}\left(\frac{T}{T}\right)Y_{T-1,T} \end{array}\right];$$

(12) $$\Psi_{X}^{\left(m\right)}=\left[\begin{array}{cccc} \psi_{m0}\left(\frac{2}{T}\right)X_{2,T}\; & \psi_{m1}\left(\frac{2}{T}\right)X_{2,T} & \cdots & \psi_{m,2^{m}-1}\left(\frac{2}{T}\right)X_{2,T}\\ \psi_{m0}\left(\frac{3}{T}\right)X_{3,T} & \psi_{m1}\left(\frac{3}{T}\right)X_{3,T} & \cdots & \psi_{m,2^{m}-1}\left(\frac{3}{T}\right)X_{3,T}\\ \vdots & \vdots & \ddots & \vdots \\ \psi_{m0}\left(\frac{T}{T}\right)X_{T,T} & \psi_{m1}\left(\frac{T}{T}\right)X_{T,T} & \cdots & \psi_{m,2^{m}-1}\left(\frac{T}{T}\right)X_{T,T} \end{array}\right]$$

After solving for ${\hat{\beta }}^{\left(\delta_{1}\right)}$ and ${\hat{\beta }}^{\left(\omega_{0}\right)}$, they can be inserted in Equation (6).

**Figure 1:**
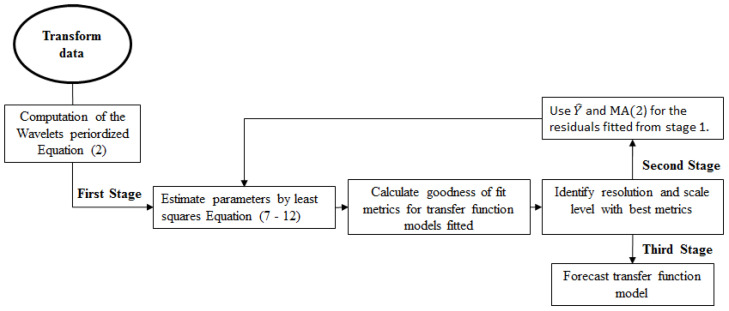
Process diagram for transfer function model with time varying coefficients.

In this paper, the models were estimated according to the methodology proposed by [17], simplifying the steps to be followed as shown in Figure 1. To obtain the periodized wavelet $\psi_{j,k}\left(u\right)$ and $\phi_{j,k}\left(u\right)$ Equation (2), the methodology implemented by [20] is used. We estimate the empirical wavelet coefficients by least squares in two stages, as described in Figure 1. In the first stage of the process, we return $Y_{t,T}$ (the response variable) from $X_{t-j,T}$ and $Y_{t-i,T}$ (explanatory variables) Equation (1). Expanding $\delta_{i}\left(\frac{t}{T}\right)$ and $\omega_{j}\left(\frac{t}{T}\right)$ in wavelet series, we obtain Equation (13). These empirical wavelet coefficients can be estimated using a Daubechies filter as in [17] and identifying the best resolution level using skill comparison metrics as: root mean squared error (RMSE) and mean absolute error (MAE), as applied in this document, but other metrics can be implemented as in [21], where a new wavelet entropy based approach was proposed to identify the optimal model specification and construct the effective wavelet entropy based forecasting models.

(13) $$Y_{t,T}=\sum_{i=1}^{m}\left(\alpha_{0,0}^{\left(\delta_{1}\right)}\phi \left(u\right)+\sum_{j=0}^{J-1}\beta_{jk}^{\left(\delta_{1}\right)}\psi_{jk}\left(u\right)\right)Y_{t-i,T}+\sum_{j=0}^{n}\left(\alpha_{0,0}^{\left(\omega_{1}\right)}\phi \left(u\right)+\sum_{j=0}^{J-1}\sum_{k\in I_{j}}\beta_{jk}^{\left(\omega_{1}\right)}\psi_{jk}\left(u\right)\right)X_{t-j,T}+e_{t,T}$$

where we have restricted the values of *j* to a maximum scale. After obtaining estimates of the wavelet coefficients $\delta_{t}\left(B\right)$ and $\omega_{t}\left(B\right)$, we use the inverse wavelet transformation to obtain the estimates of $\hat{\delta }\left(\frac{t}{T}\right)$ and $\hat{\omega }\left(\frac{t}{T}\right)$, respectively, and with $e_{t,T}$ as the error of the regression model of the first stage Equation (14). For the coefficient $\psi_{jk}\left(u\right)$ and $\phi_{jk}\left(u\right)$, we calculated *j* matrices (nine matrices) of dimension $N\cdot 2^{J}$, for the maximum value of J, (512 × 512). That is to say, every periodized moment of the signal (t/T) is an element to be sampled and convolved with the wavelet for all the possible dyadic translations and resolutions; in our case, several Daubechies filters were implemented.

(14) $$e_{t,T}=Y_{t,T}-{\hat{Y}}_{t,T}$$

In the second stage of the process, we fit the model:

(15) $$Y=\left[\begin{array}{ccccccccc} \Phi_{{\hat{Y}}_{\left(-1\right)}} & \Psi_{{\hat{Y}}_{\left(-1\right)}}^{\left(0\right)} & \cdots & \Psi_{{\hat{Y}}_{\left(-1\right)}}^{\left(j^{*}-1\right)} & \vdots & \Phi_{X} & \Psi_{X} & \cdots & \Psi_{X}^{\left(j^{*}-1\right)} \end{array}\right]\left[\begin{array}{c} \beta^{\left(\delta_{1}\right)}\\ \cdots \\ \beta^{\left(\omega_{0}\right)} \end{array}\right]+e_{2}$$

where $e_{2}$ is the random error vector, with $e_{2;t,T}$, t=2,⋯,T. In [17], it was show that each component of $e_{2}$ follows a locally stationary moving average process of order two:

(16) $$\begin{array}{cc} e_{2;t,T} & \;=Y_{t,T}-\delta_{1}\left(t\right){\hat{Y}}_{t-1,T}-\omega_{0}\left(t\right)X_{t,T}\\ \; & \;=Y_{t,T}-\delta_{1}\left(t\right)\left[Y_{t-1,T}-e_{t-1}\right]-\omega_{0}\left(t\right)X_{t,T}\\ \; & \;=Y_{t,T}-\delta_{1}\left(t\right)Y_{t-1,T}-\omega_{0}\left(t\right)X_{t,T}+\delta_{1}\left(t\right)\epsilon_{t-1,T}-\delta_{1}^{2}\left(t\right)\epsilon_{t-2,T} \end{array}$$

and it is obtained that $e_{2;t,T}=\epsilon_{t,T}-\delta_{1}^{2}\left(t\right)\epsilon_{t-2,T}$, which is a locally stationary MA(2). In this sense, in the second stage of the process, $\epsilon_{t,T}$ of Equation (1) is replaced with MA(2) from $e_{t,T}$, and $Y_{t,T}$ is replaced with ${\hat{Y}}_{t-i,T}$ to obtain the final estimates of ${\hat{\delta }}_{i}\left(\frac{t}{T}\right)$ and ${\hat{\omega }}_{j}\left(\frac{t}{T}\right)$.

According to the methodology of [17], the mother and father wavelets periodized with the original signal are convolved, and after several algebraic operations resulting from the least squares process, we obtain the wavelet coefficients, α and β Equation (4), which are introduced in the equation of wavelet expanded series to obtain finally the $\delta_{i}\left(u\right)$ and $\omega_{j}\left(u\right)$ coefficients Equation (6). The coefficients $\delta_{i}\left(u\right)$ and $\omega_{j}\left(u\right)$ are obtained in the wavelet domain. To interpret these coefficients in the time domain and substitute in Equation (1) as the weight each explanatory variable of the model, their inverse function must be calculated, which is resolved very similarly to how the inverse of a Fourier transform is calculated, that is to say:

(17) $$\hat{\delta }\left(\frac{t}{T}\right)=\Psi_{Y}^{\left(m\right)}\delta_{i}\left(u\right)$$

(18) $$\hat{\omega }\left(\frac{t}{T}\right)=\Psi_{X}^{\left(n\right)}\omega_{j}\left(u\right)$$

Synthesizing, we estimate the empirical wavelet coefficients by least squares in two stages. In the first stage of the process, we estimate the initial residuals $e_{t,T}$ and the adjusted values of ${\hat{Y}}_{t,T}$ to obtain the final estimates of $\hat{\delta }\left(t,T\right)$ and $\hat{\omega }\left(t,T\right)$ in the second phase of the process. At each stage of the process, it must be identified under what level of resolution the error is minimized, and the model is better adjusted to the data. Given that in practice, an appropriate number of levels based on the nature of the signal is usually selected [22], we perform calculations of various goodness of fit indicators to identify which one or more resolution levels we could reconstruct of $\hat{\delta }\left(t,T\right)$ and $\hat{\omega }\left(t,T\right)$, and the calculation was applied in both phases of the process Figure 1.

### 2.3. Polynomial Transfer Function Model

When requiring the modeling of the impact of a regressive variable on a response variable over time and the regressors and the response variables are assumed as stochastic processes, the approach of the transfer function models proposed by [23] can be followed, expressed as the following lagged regression model:

(19) $$Y_{t}=\sum_{j=0}^{\infty }\alpha_{j}X_{t-j}+\eta_{t}=\alpha \left(B\right)X_{t}+\eta_{t}$$

where $X_{t}$ and $\eta_{t}$ are independent stationary processes and the weights $\alpha_{j}$ measure the impact of the past values of the input variable $X_{t}$ in $Y_{t}$. The polynomial $\alpha \left(B\right)=\sum_{j=0}^{\infty }\alpha_{i}B^{i}$ is called the transfer function, and it is a polynomial in the delay operator B such that $BX_{t}=X_{t-1}$. Its coefficients must satisfy $\sum_{j=0}^{\infty }\left|\alpha_{j}\right|<\infty$ to ensure stability. The random noise $\eta_{t}$ is assumed to be stationary and can be written in the form $\eta_{t}=\frac{\theta_{\eta }\left(B\right)}{\phi_{\eta }\left(B\right)}Z_{t}$, where $Z_{t}$ is a white noise process with variance $\sigma_{Z}^{2}$.

Box et al. [23] proposed a more parsimonious representation of the transfer function as a polynomial relation:

(20) $$\alpha \left(B\right)=\frac{\delta \left(B\right)B^{d}}{\omega \left(B\right)}Z_{t}$$

where $\delta \left(B\right)=\delta_{0}+\delta_{1}B+\cdots +\delta_{s}B^{s}$ and $\omega \left(B\right)=1-\omega_{0}+\omega_{1}B-\cdots -\omega_{r}B^{r}$ and *d* is a delay coefficient. The transfer function (s,d,r) will be determined completely by estimating the coefficients of the polynomials $\delta \left(B\right)$ and $\omega \left(B\right)$ the delay coefficient *d*. This involves estimating the vector of parameters $\left(\delta_{0},\delta_{1},\cdots ,\delta_{s},\omega_{1},\cdots ,\omega_{r}\right)$. It is possible to consider a transfer function model with two or more stochastic input variables. For two input variables $x_{1t}$ and $x_{2t}$, the model has the form:

(21) $$y_{t}=\frac{\delta_{1}\left(B\right)B^{d_{1}}}{\omega_{1}\left(B\right)}x_{1t}+\frac{\delta_{2}\left(B\right)B^{d_{2}}}{\omega_{2}\left(B\right)}x_{2t}+\eta_{t}$$

This model has a much larger number of parameters than the model Equation (19), but its adjustment procedure is similar. A sequential methodology is applied to estimate the parameters of the transfer function presented in Equation (19). The methodology begins by adjusting an autoregressive moving average (ARMA) models of order (p, q) to the input time series $x_{t}$ of the form $\phi \left(B\right)x_{t}=\Theta \left(B\right)W_{t}$, where $W_{t}$ is a white noise process with variance $\sigma_{W}^{2}$; $\phi \left(B\right)=1-\phi_{1}B-\phi_{2}B^{2}-\cdots -\phi_{p}B^{p}$ is a polynomial of order p that acts on operator B and defines the autoregressive component of the model, and $\Theta \left(B\right)=1+\theta_{1}B+\cdots +\theta_{q}B^{q}$ is a polynomial of order q that defines the moving average component. Applying the operator of the ARMA model $\frac{\phi \left(B\right)}{\Theta \left(B\right)}$ on both sides of Equation (20), we obtain:

(22) $${\tilde{y}}_{t}=\alpha \left(B\right)W_{t}+\frac{\phi \left(B\right)}{\Theta \left(B\right)}\eta_{t}=\alpha \left(B\right)W_{t}+{\tilde{\eta }}_{t}$$

where ${\tilde{y}}_{t}=\frac{\phi \left(B\right)}{\Theta \left(B\right)}y_{t}$ and $\frac{\phi \left(B\right)}{\Theta \left(B\right)}\eta_{t}={\tilde{\eta }}_{t}$. In this equation, we assume that $W_{t}$ and ${\tilde{\eta }}_{t}$ are independent, where $W_{t}$ is the pre-whitened input series $x_{t}$ and ${\tilde{y}}_{t}$ and ${\tilde{\eta }}_{t}$ are the filtered output series of $y_{t}$ and the random noise $\eta_{t}$, respectively, using the operator of the ARMA (p, q) model as a filter. It can be shown that the cross-correlation between the filtered series and the pre-whitened series $W_{t}$ is $\gamma_{{\tilde{y}}_{tW_{t}}}\left(h\right)=\sigma_{W}^{2}\alpha_{h}$; therefore, their sample values allow obtaining an approximate estimate of the coefficients of the transfer function $\alpha_{0},\alpha_{1},\cdots$[16].

Shumway and Stoffer [24] presented a sequential process to fit the transfer function model, and this procedure is applied to the data as follow:

(i) Fit an ARMA model to the input series to estimate the parameters ϕ,Θ and $\sigma_{w}^{2}$ in the specification $\phi \left(B\right)x_{t}=\Theta \left(B\right)w_{t}$. Retain ARMA coefficients for use in the next Step (ii) and the fitted residuals ${\hat{w}}_{t}$ for use in Step (iii).

(ii) Apply the operator determined in Step (i), $\hat{\phi }\left(B\right)y_{t}=\hat{\Theta }\left(B\right){\tilde{y}}_{t}$ to determine the transformed output series ${\hat{\phi }}_{\eta }\left(B\right)$ and ${\hat{\Theta }}_{\eta }\left(B\right)$.

(iii) Use the cross-correlation function between ${\tilde{y}}_{t}$ and ${\hat{w}}_{t}$ in Steps (i) and (ii) to suggest a form for the components of the polynomial $\alpha \left(B\right)=\frac{\delta \left(B\right)B^{d}}{\omega \left(B\right)}$ and the estimated time delay *d*.

(iv) Obtain $\hat{\beta }=\left({\hat{\omega }}_{1},\cdots ,{\hat{\omega }}_{r},{\hat{\delta }}_{0},\cdots ,{\hat{\delta }}_{s}\right)$ by fitting a linear regression. Retain the residuals ${\hat{u}}_{t}$ for use in Step (v).

(v) Apply the moving average transformation to the residuals ${\hat{u}}_{t}$ to find the noise series ${\tilde{\eta }}_{t}$ and fit an ARMA model to the noise, obtaining the estimated coefficients in ${\hat{\phi }}_{\eta }\left(B\right)$ and ${\hat{\Theta }}_{\eta }\left(B\right)$.

#### Model Validation Methods

The model was validated using 76 records not considered in the fitting procedure. The validation data corresponded to the period between September 2005 and December 2011. As suggested by [25], model parameterization was achieved by minimizing together the root mean squared error (RMSE) and the mean absolute error (MAE) to ensure optimal results over the prediction and maximizing the correlation coefficient (R). The commonly used RMSE quantifies the differences between predicted and observed values and thus indicates how far the forecasts are from actual data. A few major outliers in the series can skew the RMSE statistic substantially because the effect of each deviation on the RMSE is proportional to the size of the squared error.

## 3. Results and Discussion

### 3.1. Data Analysis

As detailed in the previous section, the disembarkation of two species of fish (sardines and anchovies) was selected as dependent variables, whose variability could be potentially explained from the nine climatic variables presented in Table 1. In order to work the variables at the same scale, they were anomalized and standardized; this in turn allowed us to explore model fitting results under diverse temporal patterns (seasonal and slightly stationary).

#### 3.1.1. Variable Anomaly

The first step before estimating the models was to anomalize each of the indices, as well as the disembarkation of anchovy. This implies subtracting from each of the data the average monthly value calculated during a reference period and then dividing it by the monthly standard deviation (both calculated over a base period). To do so and because the World Meteorological Organization (WMO) states that a period of at least 30 years should be used for the anomaly calculation, the reference period from 1963 to 2011 was considered adequate. In other words:

(23) $$X_{ij}=\frac{X_{ij}-\bar{X_{i}}}{\sigma_{i}},i=month,j=year,$$

where the mean is given by $\bar{X_{i}}=\sum_{j=1963}^{2011}\frac{X_{ij}}{49}$ and the variance $\sigma_{i}^{2}=\sum_{j=1963}^{2011}\frac{{\left(X_{ij}-\bar{X_{i}}\right)}^{2}}{48}$.

The series of sardine landings was not anomalized because their cyclic variation is not determined by a monthly base period. To stabilize the series, a logarithmic transformation was applied.

#### 3.1.2. Variable Standardization

The standardization consisted of subtracting from each of the data the average value calculated in a reference period and then dividing it by the standard deviation (both calculated over a base period). In other words:

(24) $$X_{i}=\frac{X_{i}-\bar{X}}{\sigma },i=month,$$

where the mean is given by $\bar{X_{i}}=\sum_{j=1963}^{2011}\frac{X_{ij}}{588}$ and the variance $\sigma^{2}=\sum_{j=1963}^{2011}\frac{{\left(X_{i}-\bar{X}\right)}^{2}}{587}$.

**Figure 2:**
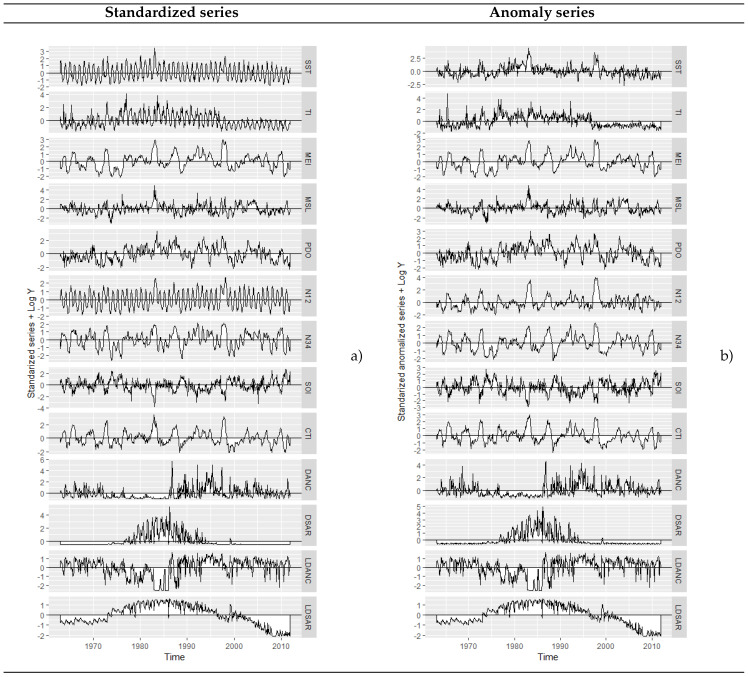
Standardized and standardized anomalies monthly series: (**a**) exogenous and response series standardized; (**b**) standardized anomalies of exogenous series and response series standardized.

The first step in any analysis and forecast of time series is to plot the observations against time, to get an idea of the possible trends and/or cycles associated with the temporal evolution of the datasets [25]. In Figure 2, it can be seen that the standardized series had periodic variation, while the exogenous variables did not seem to show periodic variation when they were anomalized; also see that the fish landings stabilized slightly when their logarithmic transformations was standardized.

**Figure 3:**
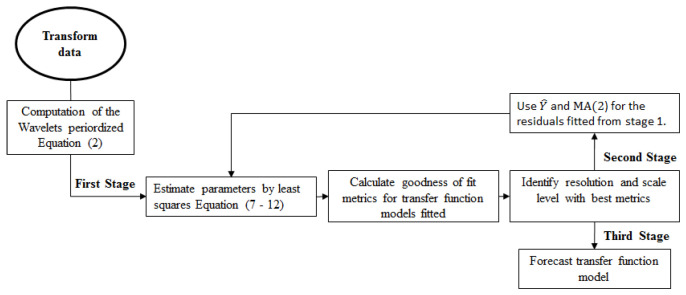
Process diagram for transfer function modeling.

Figure 3 describes the process to build the transfer functions. The raw data were divided into training and test data. The training data started from January 1963 to August 2005 (totaling 512 records), while the test data started from September 2005 to December 2011 (76 records). The different transformations (logarithmic, anomaly, standardize) were applied to the data partitions, and the transfer function models were fitted (training data). Fish landing forecasts (test data) and goodness of fit metrics were calculated for both the fitted and the forecast values. Finally, the metrics were compared, and the best model was identified.

### 3.2. Validation Results and Time Series Predictability

Table 2 shows the variables that had a significant cross-correlation with fish landings. From this crossing, the transfer function models began to be built. Based on the cross-correlation function between the fisheries’ landings and macro-climatic phenomena (Step iii, Section 2.3), correlation patterns among the series were detected; also see the variables that had the greatest linear association with the monthly anchovy and sardine disembarkation in northern Chile. These were SST,turbulence index (TI), and Niño Zone 1 + 2 (N12). Likewise, the variables with the lowest linear association were the Pacific Decadal Oscillation index (PDO), El Niño multivariate Southern Oscillation Index (MEI), Southern Oscillation index (SOI), and N34 (highly associated with N12).

It should be noted that there is a wide range of methods to identify significant variables and associated lags, such as those shown in [26]; however in the document, the methodology suggested by [24] and specified in the previous section was used (Step iii, Section 2.3).

#### Wavelet Transfer Function Models

Table 3 presents a summary of the main models obtained, with their indicators of the goodness of fit and forecast. Synthesizing, a total of 31 combinations of resolution levels was considered for ten Daubechies filters in the first stage. In the second stage, we worked with the residuals, fitted values, and the filter selected from the first stage and 31 resolution combinations. A total 310 models was fitted for each desired transfer function, in order to select the best model under diverse goodness of fit criteria.

The results shown in Table 3 allowed us to identify that through the transfer functions of variable coefficients, we could fit both anomalized and standardized data with a good level of accuracy; however, its performance was much better when the signals were standardized. Likewise, it can be observed that the models continued showing a good behavior in their residuals for the forecast phase, preserving a moderate percentage of strength in their coefficient of determination (calculated exclusively for the data based on the forecast).

We worked with each level *j* independently, as well as together until reaching an optimum level in the fitting process. In principle, it was observed for each Daubechies filter and under each combination of *j* that the mean absolute error (MAE) was minimized or the coefficient of determination $\left(R^{2}\right)$ was optimized. One might think that if the model fit were good enough, the behavior should reflect simultaneously, an elevated $\left(R^{2}\right)$ for the minimum MAE. However, this is not always the case, and this is because the wave at a specific *j* level can have the pattern of the behavior of the original signal, but not be at the scale of the same; therefore, the error could be large like $\left(R^{2}\right)$. Therefore, the best possible model for each Daubechies filter was identified, and the second stage of the process was executed, by tracking the metric goodness of fit again in the second stage, in order to obtain the best fit.

Similarly, the goodness of fit statistics were calculated, but more indicators were added to make sure that the most appropriate decision was made. In this last adjustment, the square root of the average value of the squared residuals (RMSE), MAE, the coefficient of determination $\left(R^{2}\right)$, the Pearson correlation coefficient, the Spearman correlation coefficient were calculated, as well as the Kendall correlation coefficient, all for the Daubechies filter selected in Phase 1, and in the same way for various resolution levels (j).

**Figure 4:**
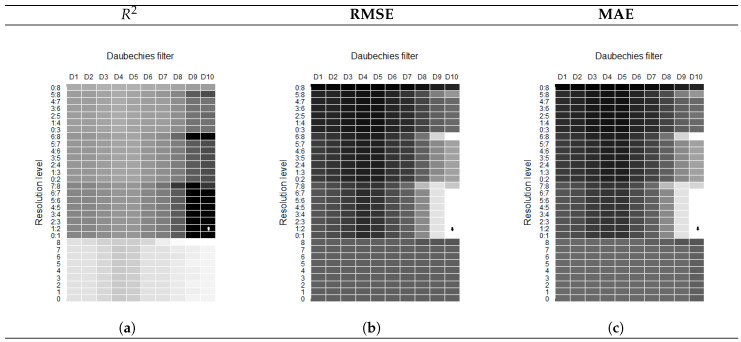
Goodness of fit metrics for the best wavelet transfer function models fitted to DANC: (**a**) Coefficient of determination $\left(R^{2}\right)$. Maximum values are identified (Max in D10, resolution 1:2). (**b**) Root mean squared error (RMSE). Minimum values are identified (Min in D10, resolution 1:2). (**c**) Mean absolute error (MAE). Minimum values are identified (Min in D10, Resolution 1:2).

For example, in Figure 4, we show the goodness of fit metrics for wavelet transfer function models estimated to explain anchovy landings (standardized data). Traditional metrics were used to determine the optimal families of wavelets and the decomposition scale, which would produce an improved forecasting performance, so the filter and resolution were selected to achieve the best metrics (high $R^{2}$ and minimum residuals). The same procedure was performed for all fitted models. The wavelet entropy algorithm such as the one presented in [21] could be used in the future, to determine the optimal wavelet families and the decomposition scale that would produce an improved forecasting performance.

### 3.3. Constant Coefficient Transfer Function Models

Table 3 presents a summary of the models obtained, with their indicators of the goodness of fit and forecast. The results shown allowed us to identify that the transfer functions of constant coefficients can model both anomalized and standardized data with a good level of fit; however, its performance was much better when the signals were standardized. Likewise, it can be observed that the models continued showing a good behavior in their residuals for the forecast phase, conserving a moderate percentage of strength in their coefficient of determination (calculated exclusively for the data based on the forecast).

### 3.4. Comparison between Transfer Function Modeling Approaches

When compared to the methodologies implemented, wavelet and polynomial coefficients with the different transformations applied to the data, in order to select the most accurate model in terms of residuals, it was observed that when the series were standardized to explain the anchovies’ landings, the transfer function wavelet model showed a better fit, while when the exogenous series were anomalized to explain the standardized log(sardine landing), the transfer function polynomial model showed a better fit. In this sense, we must keep in mind that it is convenient for the data to have certain properties so that each approach is optimal. When using constant coefficient transfer functions, it is recommended that the series have a more stable variance, which is achieved with the anomalization; while when using the variable coefficient transfer functions, the high and low frequency components of the seasonal series are captured in such a way that the models perform better.

**Figure 5:**
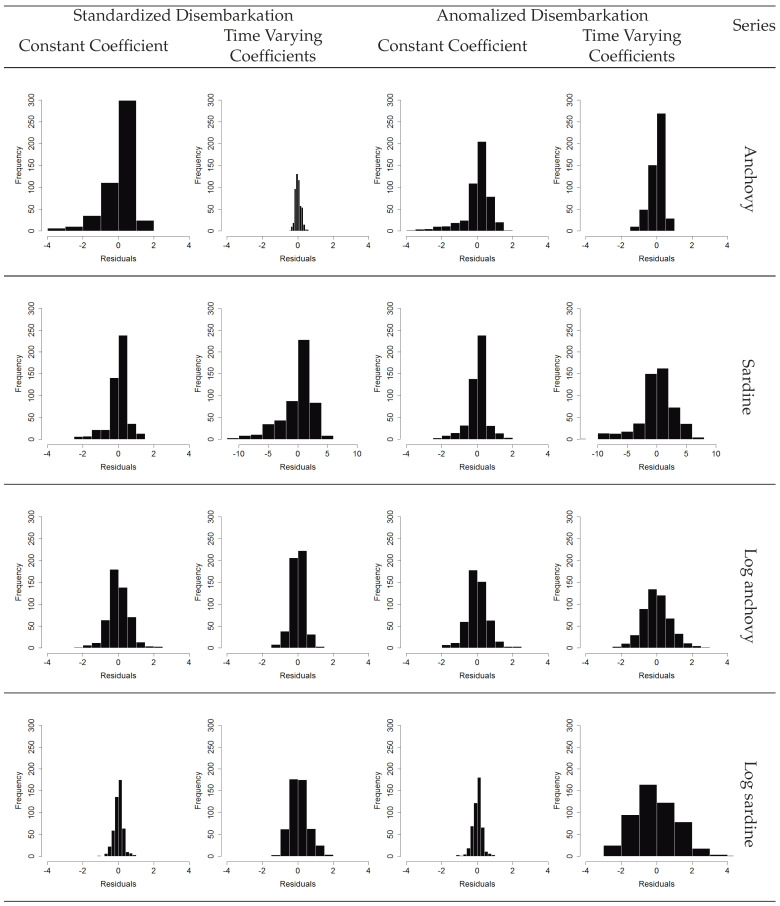
Estimated residuals between observed values and fitted values for transfer function model methods.

**Figure 6:**
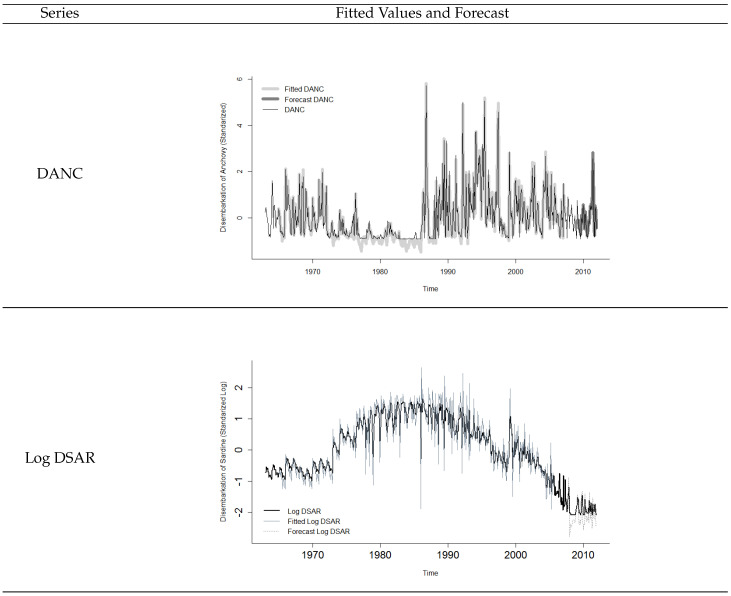
Standardized and scaled anomalized monthly series for anchovies landings (**top**) and sardine landings in logarithmic scale (**bottom**).

Figure 5 shows the residuals of the fitted models, for different treatments of the variables and transfer function models. It was verified that the best models (with smaller residuals) were obtained when using a transfer function with variable coefficients to explain the anchovies’ landings (data standardized); and when using a transfer function with polynomial coefficients to explain the sardines landings (explanatory variables anomalized and sardine series in standardized logarithmic scale). Figure 6 shows the fit behavior of both models, as well as the forecasts obtained from the test data.

Likewise, we can see that the variance explained during the validation phase for anchovy landings (96,9%) showed important improvements in relation to previous works, such as that presented by [7], where the variance explained in external validation fluctuated between 84% and 87%, or in sardine and anchovy landing forecasts presented by [9], in which the variance explained by both models was slightly higher than 82%.

We can observe that the sardine landings did not reach as good a fit as those of anchovies. This could be optimized in a future work by considering, for example, a truncated model as suggested by [27], where the data series were modeled based on the assumption that the data followed a truncated and transformed multivariate normal distribution. In their work, the data predictive inferences showed very realistic results, capturing the typical variability of the series in time and space.

## 4. Conclusions

Based on the analysis of models built to forecast the monthly disembarkation of anchovy (*Engraulis ringens*) and sardine (*Sardinops sagax*) in northern Chile, the following conclusions emerged from the analysis:

When various transformations were applied to the data to achieve better model precision, large differences in the benefits of the selected fitted models could be identified. Records of anchovy landings were better fitted and forecast with standardized data under a transfer model with wavelet coefficients, using Daubechies 10 filters with low resolution levels (associated with the slightly compressed wavelets j=1:2). Sardine landings were better fitted when the variance of the landings was stabilized using the logarithmic function; and the variance of the explanatory variables was stabilized by anomalizing the variables; finally, modeling these sardine landings in a logarithmic scale with a traditional transfer function.

The variables that allowed explaining in a more robust way the disembarkation of anchovy was the turbulence index from Antofagasta Coastal Oceanographic Station (TI) and the Pacific sea surface temperature index (Niño Zone 1 + 2: N12); while the disembarkation of sardines was explained by local climatic variables: TI, sea surface temperature from Antofagasta Coastal Oceanographic Station (SST), and the log disembarkation of anchovy.

Given that the process of selecting the appropriate number of scales to optimize the model fit was made according to the researcher’s choice, it is advisable to implement in the future some entropy based techniques that allow for the best possible scale selection. It is also recommended to evaluate if the results can be optimized considering other wavelet filters in addition to the Daubechies filters. Likewise, a non-linear structure model could be considered (for example, thresholding wavelet coefficients) in order to determine the best model structure for fishery prediction. These results could also be optimized by implementing bootstrapping techniques for the fitted parameters in order to quantify their uncertainty.

## Figures and Tables

**Table 1:** Variable that are the object of study. The climatic variables are explanatory, while local fisheries are the response variables. All records are monthly from 1963 to 2011.

Type	Variable	
	SST	Sea surface temperature from Antofagasta Coastal Oceanographic Station
Local Climatic	TI	Turbulence index from Antofagasta Coastal Oceanographic Station
	MSL	Mean sea level from Antofagasta Coastal Oceanographic Station
Global Climatic	MEI	El Niño multivariate Southern Oscillation index
	PDO	Pacific Decadal Oscillation index
	N12	Pacific sea surface temperature index (Niño Zone 1 + 2)
	N34	Pacific sea surface temperature index (Niño Zone 3 + 4)
	SOI	Southern Oscillation index
	CTI	Cold tongue index
Local Fisheries	DANC	Disembarkation anchovy (*Engraulis ringens*) in northern Chile
	DSAR	Disembarkation sardine (*Sardinops sagax*) in northern Chile

**Table 2:** Cross correlation coefficients (CCF) for different lags between disembarkation and significant explanatory variables.

X	Standardized				Anomalized				Standardized				Anomalized			
	Anchovy		Log Anchovy		Anchovy		Log Anchovy		Sardine		Log Sardine		Sardine		Log Sardine	
	Lag	CCF	Lag	CCF	Lag	CCF	Lag	CCF	Lag	CCF	Lag	CCF	Lag	CCF	Lag	CCF
SST	-	-	-	-	15	−0.10	7	−0.13	-	-	20	0.22	15	0.13	15	0.12
TI	2	−0.12	-	-	2	−0.14	10	−0.20	18	0.15	18	0.23	2	0.14	18	0.22
MEI	-	-	-	-	-	-	5	0.11	10	0.15	-	-	-	-	-	-
MSL	-	-	7	−0.15	3	−0.11	7	−0.15	-	-	-	-	3	0.08	-	-
PDO	-	-	-	-	-	-	-	-	-	-	2	0.10	-	-	-	-
N12	22	−0.11	5	−0.12	-	-	5	−0.11	-	-	24	0.14	-	-	-	-
N34	-	-	-	-	-	-	-	-	-	-	-	-	-	-	-	-
SOI	-	-	-	-	-	-	-	-	23	−0.11	-	-	-	-	-	-
CTI	-	-	-	-	-	-	-	-	12	−0.11	22	0.11	-	-	-	-
DSAR	-	-	5	−0.19	-	-	5	−0.19	1	0.80	1	0.95	1	0.80	1	0.95
DANC	1	0.54	1	0.76	1	0.57	1	0.76	10	−0.15	7	−0.14	-	-	7	−0.14

**Table 3:** Transfer function models. Goodness of fit indicators for the resulting models. In bold are the results of the best models for each time series.

T${}^{1/}$	Y${}^{2/}$	X${}^{3/}$	L${}^{4/}$	Type Coefficient	Fitted						Forecast		
					RMSE	MAE	R${}^{2}$	Pearson	Spearman	Kendall	RMSE	MAE	R${}^{2}$
Anchovy													
N	**DANC**	**DANC**	**1**	Constant	0.851	0.559	0.821	0.964	0.882	0.727	0.603	0.464	0.796
		**TI**	**2**										
		**N12**	**22**	**Wavelet**	**0.165**	**0.128**	**0.978**	**0.990**	**0.971**	**0.865**	**0.138**	**0.106**	**0.969**
A	DANC	DANC	1	Constant	0.831	0.568	0.826	0.966	0.919	0.771	0.660	0.571	0.750
		SST	15										
		TI	2	Wavelet	0.416	0.309	0.904	0.956	0.943	0.796	0.265	0.207	0.824
		MSL	3										
Log N	DANC	DANC	1	Constant	0.603	0.451	0.862	0.953	0.950	0.813	0.770	0.575	0.748
		MSL	7										
		N12	5	Wavelet	0.391	0.286	0.883	0.932	0.929	0.775	0.717	0.806	0.564
		LDSAR	5										
Log A	DANC	DANC	1	Constant	0.604	0.447	0.860	0.950	0.948	0.808	0.792	0.548	0.751
		SST	7										
		TI	10										
		MEI	5	Wavelet	0.818	0.620	0.652	0.714	0.637	0.458	0.599	0.493	0.681
		MSL	7										
		N12	5										
		LDSAR	5										
Sardine													
N	DSAR	DSAR	1	Constant	0.610	0.380	0.858	0.943	0.640	0.472	0.167	0.135	0.500
		TI	18										
		MEI	10										
		SOI	23	Wavelet	1.033	0.734	0.672	0.715	0.538	0.374	1.308	1.072	0.500
		CTI	12										
		DANC	10										
A	DSAR	DSAR	1	Constant	0.614	0.381	0.859	0.947	0.744	0.584	0.139	0.109	0.619
		SST	13										
		TI	12										
		SOI	23	Wavelet	1.377	0.998	0.590	0.557	0.290	0.192	0.887	0.749	0.499
		CTI	18										
Log N	DSAR	DSAR	1	Constant	0.274	0.199	0.918	0.962	0.966	0.840	0.303	0.258	0.688
		SST	20										
		TI	18										
		PDO	2	Wavelet	1.013	1.261	0.613	0.614	0.607	0.432	0.357	0.285	0.547
		N12	24										
		CTI	22										
		LDANC	7										
**Log A**	**DSAR**	**DSAR**	**1**	**Constant**	**0.276**	**0.200**	**0.916**	**0.961**	**0.966**	**0.840**	**0.277**	**0.239**	**0.706**
		**SST**	**15**										
		**TI**	**18**	Wavelet	0.417	0.542	0.562	0.755	0.762	0.565	1.291	1.249	0.502
		**LDANC**	**7**										

${}^{1/}$ Type of transformation made to the variables: standardized (N), anomalized (A). ${}^{2/}$ Response variable (Y). ${}^{3/}$ Explanatory variable (X). ${}^{4/}$ Lag of the explanatory variable in the transfer model (L=Lag).
